# Characterization of DNA G-Quadruplex Structures in Human Immunoglobulin Heavy Variable (IGHV) Genes

**DOI:** 10.3389/fimmu.2021.671944

**Published:** 2021-05-10

**Authors:** Catherine Tang, Thomas MacCarthy

**Affiliations:** ^1^ Department of Applied Mathematics and Statistics, Stony Brook University, Stony Brook, NY, United States; ^2^ Laufer Center for Physical and Quantitative Biology, Stony Brook University, Stony Brook, NY, United States

**Keywords:** immunoglobulin heavy chain (Igh), somatic hypermutation (SHM), activation induced deaminase (AID), G-quadruplex, IGHV genes

## Abstract

Activation-induced deaminase (AID) is a key enzyme involved in antibody diversification by initiating somatic hypermutation (SHM) and class-switch recombination (CSR) of the Immunoglobulin (Ig) loci. AID preferentially targets WRC (W=A/T, R=A/G) hotspot motifs and avoids SYC (S=C/G, Y=C/T) coldspots. G-quadruplex (G4) structures are four-stranded DNA secondary structures with key functions in transcription, translation and replication. *In vitro* studies have shown G4s to form and bind AID in Ig switch (S) regions. Alterations in the gene encoding AID can further disrupt AID-G4 binding and reduce CSR *in vivo*. However, it is still unclear whether G4s form in the variable (V) region, or how they may affect SHM. To assess the possibility of G4 formation in human V regions, we analyzed germline human Ig heavy chain V (IGHV) sequences, using a pre-trained deep learning model that predicts G4 potential. This revealed that many genes from the IGHV3 and IGHV4 families are predicted to have high G4 potential in the top and bottom strand, respectively. Different IGHV alleles also showed variability in G4 potential. Using a high-resolution (G4-seq) dataset of biochemically confirmed potential G4s in IGHV genes, we validated our computational predictions. G4-seq also revealed variation between S and V regions in the distribution of potential G4s, with the V region having overall reduced G4 abundance compared to the S region. The density of AGCT motifs, where two AGC hotspots overlap on both strands, was roughly 2.6-fold greater in the V region than the Constant (C) region, which does not mutate despite having predicted G4s at similar levels. However, AGCT motifs in both V and C regions were less abundant than in S regions. *In silico* mutagenesis experiments showed that G4 potentials were generally robust to mutation, although large deviations from germline states were found, mostly in framework regions. G4 potential is also associated with higher mutability of certain WRC hotspots on the same strand. In addition, CCC coldspots opposite a predicted G4 were shown to be targeted significantly more for mutation. Our overall assessment reveals plausible evidence of functional G4s forming in the Ig V region.

## Introduction

Antibody diversification in antigen-stimulated B cells is instrumental in driving effective humoral immunity against a wide range of pathogens. Activation-induced deaminase (AID) is a critical enzyme responsible for initiating somatic hypermutation (SHM) and class-switch recombination (CSR) at the Immunoglobulin (Ig) loci [reviewed in (1)]. There are ~55 Ig heavy chain variable (IGHV) genes in human, each of which may have one or more alleles. The IGHV genes are further categorized into families, defined by sequence similarity (2). AID binds to single-stranded DNA (ssDNA) and deaminates cytosine (C) to uracil (U). During SHM, co-opted DNA repair pathways, including base-excision repair and mismatch repair, result in unfaithfully repaired AID-induced lesions. Ig variable (V) region SHM occurs in the dark zone of the germinal center (GC) at a rate of around 10^-3^ per bp per cell division. In the GC light zone, positive selection of B cells that acquire mutations, leading to higher binding affinity of the B cell receptor, takes place. During CSR, AID mutations lead to DNA double-stranded breaks (DSBs) in the Ig switch (S) region. Class switching is achieved when the IgM heavy chain constant (C) region of the antibody is swapped for another one of the downstream isotypes, thus changing the effector function of the antibody while maintaining antigen specificity.

Certain DNA sequence contexts are mutated preferentially by AID, and are collectively referred to as “hotspots” (3). The canonical AID hotspot is defined as WRC (W=A/T, R=A/G; and the underlined base indicates the site targeted). In addition, the AGCT motif, a palindromic variation of WRC, is enriched in the S region (4) and is targeted at particularly high rates in the V region as well (5). Conversely, the SYC (S=C/G, Y=C/T) “coldspot” is targeted significantly less by AID (6).

DNA secondary structures play an active role in regulating the transcriptional process. The G-quadruplex (G4) is a four-stranded nucleic acid structure formed from G-rich DNA. Hoogsteen base-pairing allows four G nucleotides to associate and form a planar G-tetrad, and the stacking of multiple G-tetrads results in a stable G4 structure. G4s can further be stabilized in the presence of a monovalent cation, such as K^+^ or Na^+^, or by interacting with a small molecule such as pyridostatin [PDS (7, 8)]. Several computational methods have been developed to detect putative G4s in DNA and RNA sequences (9). Early models were designed to identify canonical G4s by simple string matching, for example, searching for four G-repeats each with a length of at least three, interspersed by loops no longer than 7 nt in length (10). Later studies discovered that G4s could adopt more complicated conformations, such as containing longer loops, bulges or mismatches, as well as two tetrad structures, thereby leading to newer methods adapting to these alternative structures. These non-canonical conformations were later validated with the emergence of G4-seq, a high-throughput sequencing technique designed for genome-wide G4 detection (11). Identifying G4 structures in chromatin was also made possible by utilizing chromatin immunoprecipitation and high-throughput sequencing (ChIP-seq) with an antibody-based G4 [G4 ChIP-Seq (12)]. All together, these high-throughput methods provided a way of curating large, high-resolution datasets, which eventually enabled more powerful and accurate machine learning G4 detection algorithms to be devised.

Several previous studies have shown G4s to be actively forming in the Ig loci. One key study discovered G4s mimicking S regions to be a preferred target for AID *in vitro*, and that these structured substrates induced AID oligomerization upon binding (13). Earlier work also found that AID can bind to transcription-induced G4s in the S region as well as certain proto-oncogenes (e.g. c-MYC and BCL6), suggesting G4s might be involved in recruiting AID to these regions (14, 15). RNA G4s are formed during S region transcription and facilitate the creation of R loops leading to CSR (16). Recent work has shown that the G133V mutation in the AID-encoding gene seen in hyper-IgM patients prevents AID from localizing to the S region (13, 17). An *in vivo* investigation of mice bearing this critical mutation noted that AID-G4 binding was also compromised, which subsequently led to a reduction in both CSR and SHM. Strikingly, SHM in the homozygous mutant mice was reduced to the same level as AID homozygous KO, as measured by mutations in the Vh558-Jh4 intron (17). These results further implicate G4 structures in AID targeting for both CSR and SHM.

Thus far, little work has been done to elucidate the role of G4 structures in the V region repertoire, particularly in human. In addition, the association of G4s in SHM has only been measured indirectly by using, for example, the mouse Jh4 intron as a proxy for measuring SHM, rather than considering mutations within the rearranged V(D)J coding exons (17). We, therefore, sought a comprehensive evaluation of G4 assembly in human IGHV regions, as well as the possible impact G4s may have on SHM. In this study, we searched for evidence of G4 formation in 232 functional human IGHV alleles using a deep learning model, *G4detector*, that computes the potential for forming a G4 of a given DNA sequence. This approach identified high-probability G4s particularly in the IGHV3 and IGHV4 families. Furthermore, there is a good correspondence between the computational G4 predictions and experimentally derived (G4-Seq) G4 structures. Increased G4 potentials are also found to be associated with increased targeting at certain AID hotspots. We further performed *in silico* mutagenesis experiments and assessed the impact hypothetical mutations might have on G4 potential, finding that G4s are largely robust to mutation, although certain subregions are vulnerable, particularly in framework (FW) regions. Mutational targeting to CCC coldspot motifs is increased when they occur on the opposite strand to a GGG motif predicted to participate in G4 formation.

## Methods

### Germline IGHV Data

The reference human germline IGHV sequences used in this analysis were downloaded from the international ImMunoGeneTics information system (IMGT) last accessed on December 1, 2020 (2). In addition, we utilized only functional alleles and removed any sequences with partial 5’ or 3’ regions, resulting in a total of 232 unique alleles across the seven IGHV families (IGHV1-7). The IMGT unique numbering system was also utilized to distinguish framework (FW) and complementarity determining region (CDR) boundaries.

### Calculating G4 Potentials

We utilized *G4detector* (https://github.com/OrensteinLab/G4detector), a deep learning model that uses a multi-kernel convolutional neural network (CNN) framework, to calculate the G4 potentials of the IGHV alleles (18). *G4detector* takes a linear DNA sequence of fixed length (297bp) and outputs a single continuous number between 0 and 1 that is an estimate of the potential for that sequence to form a G4. The h5 file containing the CNN model was downloaded and used with Python. Each input sequence was converted to its 1-D one-hot encoding (i.e. a string of zeros and ones) of length 1188 (297 x 4 DNA bases). In other words, every four positions of the vector - each position representing one of the four ordered DNA bases - signifies a single nt position in the original DNA sequence, and a one indicates the base contained in the original sequence. In the event where the input sequence was shorter than 297bp, zeros were padded at the end of its respective one-hot encoding. Conversely, if the sequence was longer than the allowable length, then it was truncated to 297bp before being converted to one-hot format.

### Computing and Visualizing Integrated Gradients

Custom Python scripts were written to compute the integrated gradients of *G4detector* model predictions. The integrated gradients method works by taking the straight-line path integral from a baseline input to the actual input (19). For our study, we used a 0-matrix as our baseline input. Sequence feature attributes of the corresponding integrated gradients were visualized using Logomaker (20).

### Finding the Correlation Between G4 Potentials and G4-Seq Data in Human Reference Genome Genes

We first used string matching to find each IGHV allele that was aligned to the human reference genome (hg19). In total, there were 40 functional IGHV genes identifiable in the genome. Previously published G4-seq data (11) containing the locations of genome-wide G4s were downloaded from the Gene Expression Omnibus (accession number: GSE63874). The G4-Seq assay of Chambers et al. quantifies the presence of G4s by measuring the percentage of read mismatches induced by polymerase stalling in the presence of G4-promoting agents during the sequencing process. We then found the maximum percent mismatch that was reported within each gene. Spearman correlations were subsequently calculated between the maximum percent mismatch and corresponding *G4detector* prediction for each gene.

### *In Silico* G4 Experiments

Simulations were performed to assess G4 outcomes in different mutational scenarios. In the first instance, we hypothesized that substituting an individual G at a non-G site would increase the G4 potential from its germline reference. For each IGHV allele, we created as many new sequences as there were total A, C, and T bases found within the germline context. In other words, each newly generated mutated sequence contained a single H>G (H=A/C/T) point mutation. The G4 potentials of all ensuing mutated sequences were then assessed using *G4detector*. A second simulation was performed in a similar fashion, except in this case, we mutated away G’s one site at a time. In addition, for every G site, we created three newly mutated sequences for each mutational outcome (i.e. G>H), ran them through *G4detector*, and then took the average of the G4 potentials. Differences in G4 potentials were calculated by subtracting the observed potential of the germline subject from its corresponding mutated predictions.

### Obtaining and Visualizing IGH and G4-Seq Genome Browser Tracks

Tiled data files (tdf) and BED files containing observed G4s subjected to PDS stabilizing conditions were also downloaded from the Gene Expression Omnibus (accession number: GSE63874) and analyzed in this study. The BED file containing the locations of IGH constant genes were downloaded from the Genome Reference Consortium (https://www.ncbi.nlm.nih.gov/grc/human/regions/IGH?asm=GRCh37.p13). Previously mapped IGHV gene locations were written to a BED file as well. All tracks were aligned to the human reference genome (hg19) and displayed using the Integrative Genomics Viewer (IGV) desktop interactive tool (21).

### Building IGHV Consensus Sequences From the G4-Seq Data

We first downloaded the fasta file containing chr14 of the hg19 genome from hgdownload.cse.ucsc.edu/goldenPath/hg19/chromosomes/chr14.fa.gz. The fastq file containing one of the two Read 1 (control condition) of the original G4-seq data was obtained using the Sequence Read Archive (SRA) Toolkit (accession: SRR1693710), and subsequently aligned to chr14 using the bwa “mem” algorithm with default parameters as outlined by Chambers et al. Bcftools (https://samtools.github.io/bcftools/) was utilized to generate genotype likelihoods at each genomic position with coverage (bcftools mpileup), as well as to detect all possible variants (bcftools call) from the alignment step. The consensus sequence was built (bcftools consensus) from the subsequent VCF file, using the reference genome as the default if multiple alleles were detected. Individual consensus sequences for each IGHV gene were extracted using the genomic coordinates contained in the above-mentioned BED file (see previous section). Lastly, variation observed due to SHM was calculated as the number of nucleotides attributed to non-allelic variants divided by the total read coverage of the IGHV genes.

## Results

### The Potential to Form G4s Varies Between IGHV Genes and Alleles

Several studies have identified G4s in Ig S regions (13, 22). However, directly verifying AID-G4 binding *in vivo* remains elusive. Adapting next-generation sequencing technology to find G4 structures genome-wide (G4-seq) has made large amounts of data available (11), facilitating more advanced methods to computationally predict G4s using linear DNA sequences. Here, we used a previously published deep learning model, called *G4detector* (18) (https://github.com/OrensteinLab/G4detector), to assess potential G4s within the IGHV alleles (see *Methods*). *G4detector* was developed using high-resolution G4-seq data from both *in vitro* (G4-Seq) and *in vivo* (G4 ChIP-Seq) experiments, thus enabling the model to predict G4 formation across many different genomic contexts. The use of deep learning also makes *G4detector* more accurate than previous G4 prediction methods, many of which are dependent on simple string pattern matching for subregions that align with a pattern, such as G_3+_N_1-7_G_3+_N_1-7_G_3+_N_1-7_G_3+_ (where G_3+_ represents a run of at least 3 consecutive G’s and N=A/C/G/T) as a consensus G4 sequence (23–26), but this approach can give many false positives.

**Figure 1:**
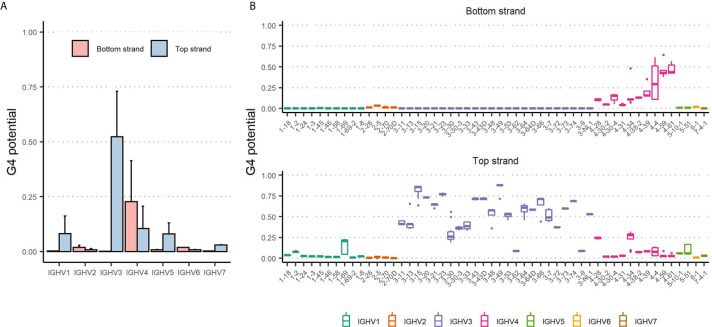
G4 potential of the human IGHV alleles. **(A)** Mean G4 potentials of 232 functional IGHV alleles grouped by IGHV family are shown separately for bottom strand (red) and top strand (blue). Error bars representing one standard deviation are shown in black above each bar. **(B)** Box plots displaying G4 potentials grouped by IGHV gene. Individual IGHV families are distinguished by different colors.

Analysis of the IGHV alleles using *G4detector* shows a wide range of G4 outcomes, including disparities observed between the top and bottom strands (**Figure 1A**). Overall, the top strand displayed higher G4 potentials (predicted probability range: 0-0.89; mean: 0.27 ± 0.27) than the bottom strand (range: 0-0.64; mean: 0.051 ± 0.13). The contrast in potentials between the two strands aligns with evidence that G4s are disproportionately favored to form on the coding strand (27). If we considered IGHV families separately, we found that the IGHV3 family had the highest propensity to form G4s on the top strand (mean: 0.52 ± 0.21), whereas the IGHV4 family showed the strongest preference to form bottom strand G4s (mean: 0.23 ± 0.19). Altogether, our computational assessment indicates considerable heterogeneity amongst the 7 human V region families, which are composed of very different numbers of genes, and that some V regions, especially in the IGHV3 family, have a high predicted probability of forming G4s.

In addition, we further noted differences in G4 potentials between alleles stemming from the same IGHV gene (**Figure 1B**). For example, the IGHV4-4 gene contains four alleles whose bottom strand potentials range from 0.10 to 0.62. To give another example, in the IGHV3 family, the IGHV3-49 gene contains one allele (IGHV3-49*02) with a G4 potential of 0.72, and the remaining four alleles having values of 0.88 or greater.

Since *G4detector* does not reveal the G4 structure itself, we needed a way to ascribe our G4 predictions to relevant features of the input sequence. One challenge of deep learning - often labelled as the “black box problem” - is the interpretability of a model. Several methods have been developed as a way to conceptualize a model’s output with respect to a given input. As such, we have chosen to use integrated gradients as our method of interpreting *G4detector* outcomes (see *Methods*). This process enables us to quantify the important sites within the input sequence that contributed highly to the model’s G4 prediction and, moreover, allows us to infer the regions involved in G4 assembly. As one would expect, runs of G nucleotides are the dominant contributing feature identified by this method (**Supplementary Figure 1**). In the case of IGHV3-49*02, integrated gradients identified one run of G’s that was disrupted (by having a C instead of a G at IMGT position 50), but which remained intact in the other IGHV3-49 alleles. However, when we made a C>G substitution at IMGT position 50 to restore the tandem G’s, the G4 potential in IGHV3-49*02 was elevated from 0.72 to 0.87 - a level similar to the other alleles (**Supplementary Figure 1**). These results suggest that many observed differences between alleles may be due to sequence variation seen in G-rich regions critically involved in G4 formation.

### G4s Assemble in the V Region *In Vitro* and Are Less Active Compared to S Regions

**Figure 2:**
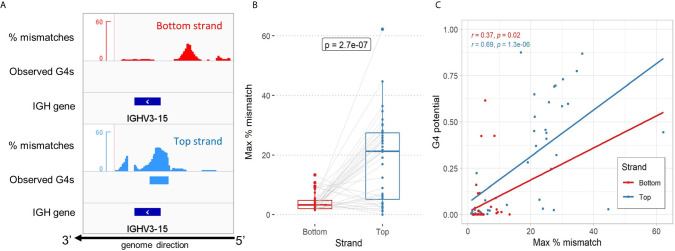
Assessment of high-throughput experimental G4-seq data in the Ig V region. **(A)** Genome browser view showing the distribution of percent mismatches according to Chambers et al. for the IGHV3-15 gene. Observed G4s indicate regions reaching a threshold of 25% or above (red and blue bars below mismatches for bottom and top strand, respectively). The gene location is shown as a dark blue bar below. All tracks are aligned to the reverse strand of the human hg19 reference genome. **(B)** Comparison of the maximum percent mismatch of the 40 IGHV genes located in the genome between the bottom strand (red) and top strand (blue). A two-sided non-parametric t-test (Mann-Whitney) comparing the two strands was performed. **(C)** Comparison between experimental G4-seq data and *G4detector* predictions by strand. Reported Spearman correlation (r) and p-value for each strand is shown in their respective color.

In order to verify the predictions made by *G4detector*, we utilized a published dataset of biochemically validated experimental G4 data based on a human genome-wide screen (G4-Seq), to look for instances of G4s forming in the V region. The G4-Seq assay of Chambers et al. (11) quantifies the presence of G4s by measuring the percentage of read mismatches induced by polymerase stalling in the presence of G4-promoting agents during the sequencing process. The authors also defined observed G4s as regions exceeding a threshold of 25% mismatches (**Figure 2A** for example of IGHV3-15). We began by assessing instances of G4s forming in the 40 functional IGHV genes we identified in the human reference genome (hg19 - see *Methods*). We found the maximum percent mismatch of a gene in the top strand to be significantly higher than in the bottom strand (p-value=2.7×10^-7^; **Figure 2B**). This result confirms our predictions and is consistent with previous evidence showing that G4s are more readily created on the coding strand [**Figure 2B**; (27)]. Furthermore, we looked to quantify the correspondence between predicted G4 potentials and the G4-seq data for all genes (see *Methods*). In this instance, we discovered there to be a significant positive correlation between G4 potentials and maximum percent mismatches in the top strand (r=0.69, p-value=1.3×10^-6^; **Figure 2C**) as well as in the bottom strand (r=0.37, p-value=0.02; **Figure 2C**). Overall, our predictions appear to be in good accord with experimental G4-seq data, especially for top strand G4s.

**Figure 3:**
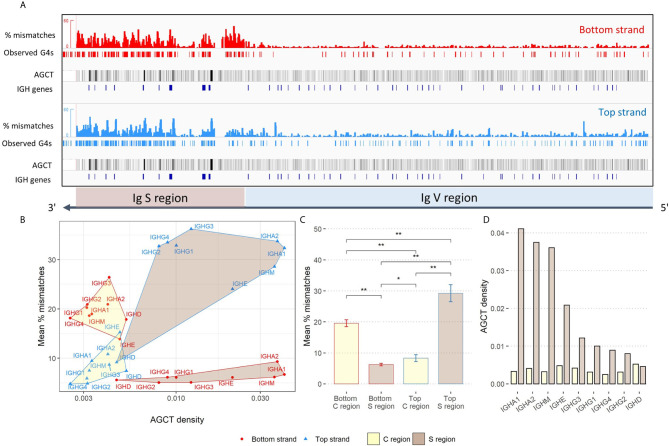
Analysis of G4 activity in the IgH locus. **(A)** Same as **Figure 2A**, except showing an expanded view of G4 activity in the Ig S and V region. AGCT motifs are represented as black tracks and are shaded using the mean windowing function in IGV. **(B)** Comparison of the density of AGCT motifs (x-axis; log-scale) and mean percent mismatches (y-axis) representing overall G4 activity within each constant (C) region (yellow shading) and S region (brown shading). Edges, vertices and labels of all regions are colored according to strand location (red: bottom; blue: top). **(C)** Quantifying G4 activity within C regions (yellow) and S regions (brown) for both bottom and top strands. Error bars above each bar represent -/+ 1 standard deviation. Significant p-values from two-sided Mann-Whitney U tests are shown in asterisks (*p ≤ 0.05; **p ≤ 0.01). **(D)** Density of AGCT motifs by isotype as observed within each C region (yellow) and S region (brown).

Additionally, we searched for instances of G4s forming in the switch (S) regions - located upstream of each Ig constant (C) region - to see how the overall G4 activity in the V region compares against the S region, where there is extensive evidence for G4 formation *in vitro* (13). For all isotypes except IgD, we qualitatively observed an enrichment of observed G4s in the top strand S region (**Supplementary Figure 2A**). In contrast, observed G4s were more sparse in the S regions in the bottom strand, although there were some regions of higher density in the C regions themselves. Our observations of experimentally derived G4s in the Ig heavy chain locus suggest that V regions tend to be much less enriched for G4s compared to S regions (**Figure 3A** **)**.

Given the differences we observed between the C and S regions, we next wanted to measure and compare the density of AGCT hotspots in these two subregions, considering the fact that the AGCT motif is known to be abundant in the S region and can facilitate CSR [See **Supplementary Figure 2B** for example of IgM; (4)]. As a starting point, we calculated the density of AGCT motifs, for every C region and upstream S region, by counting the number of instances AGCT appeared in each subregion separately, and then normalized each count by its sequence length. S regions were inspected and manually assigned boundaries upstream of the start of each C region. Additionally, we calculated G4 activity as the average percent mismatch (from the G4-Seq data) within these regions, for both strands. The top strand S regions were highest in terms of mean G4 activity, except for IgD, followed by the bottom strand C regions (**Figures 3B, C**). There were also significant differences between top and bottom strand C regions and S regions in terms of their mean G4 activity (**Figure 3C**). Additionally, there appeared to be a discrepancy between top and bottom strand C regions, with the bottom strand being more enriched for G4s than the top strand, albeit less enriched compared to top strand S regions. Again, with the exception of IgD, we further observed S regions to have a greater density of AGCT motifs compared to C regions (**Figures 3B, D**). IgD is an anomaly presumably because switching seldomly occurs there as shown in both mouse and human (28, 29). Not only is the density of AGCT motifs of the IgD the lowest amongst the other classes, but also that the density is roughly equal within both the C and S region.

We similarly compared G4s in the C region to the IGHV genes. Here, we found that bottom strand C region G4s (as measured by mean % mismatches) are significantly elevated compared to both strands of the IGHV genes (**Supplementary Figure 3A**). On the other hand, the level of G4s in the top strand of C regions is not significantly different from G4s of the top strand in the IGHV genes. We also noted approximately a 2.6-fold increase in AGCT density in the V region (9.78×10^-3^) from the C region (3.75×10^-3^; **Supplementary Figure 3B**). However, the density of AGCT motifs in the V region is still roughly 2-fold less than in the S region (2×10^-2^). Given that the C regions do not mutate, whereas the V regions do, this suggests that AID is strictly regulated to be kept away from the C regions, despite their physical proximity to S regions and their having relatively high levels of G4s. The particularly low density of AGCT sites in the C regions is also likely to be a contributing factor.

We should note that the G4-Seq data of Chambers et al. were also used to train the *G4detector* model (together with another *in vivo* dataset); however, the G4-Seq assay was performed genome-wide and since the IGHV genes represent only a very small fraction of the genome, there is little risk of these results being due to overfitting. In addition, since the G4-seq data was derived from B-cell DNA libraries, we checked that VDJ rearrangement and SHM were not having a large effect on our results. Starting with a control dataset from the original publication, we replicated the initial steps of the pipeline described by Chambers et al. [(11); see *Methods*]. After mapping to the reference genome, it was clear from visual inspection of the alignment that the mapping step removes most, if not all, sequences containing CDR3 regions, as expected. Then, after excluding any viable alternative alleles, we determined that the level of naturally occurring SHM was low, at around 1.3%. We also built consensus sequences for each IGHV gene derived from the data, and then evaluated their G4 potential using *G4detector*. We found that the G4 potentials of the G4-seq consensus sequences were very similar to those of the closest IMGT germline, with G4 potentials, on average, only differing by 0.06% and 0.3%, for the bottom and top strand, respectively (**Supplementary Tables 1A, B** for the bottom and top strand, respectively). Thus, SHM in the G4-seq data is minimal and the underlying sequences are very similar to the corresponding IMGT germline sequences.

### Targeting to AID Hotspots Is Increased With G4s Formed on the Same Strand

**Figure 4:**
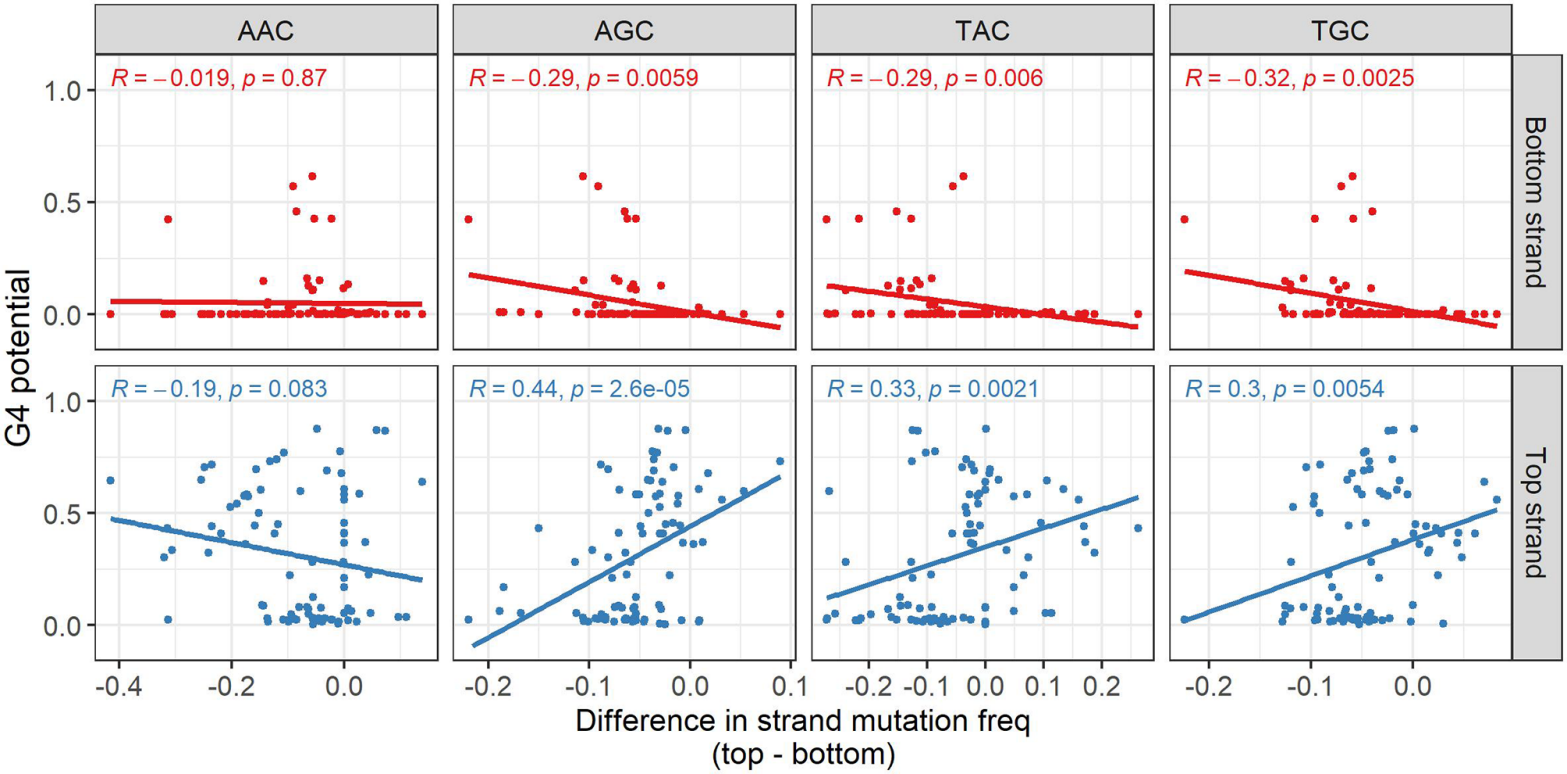
Influence of predicted G4s on AID targeting to hotspots. Association between the predicted G4 potential (y-axis) and difference (top-bottom) in mutation frequency of the various AID hotspot motifs. For each gene, the difference in the average mutation frequency of the hotspot motif on the bottom strand from the corresponding hotspot on the top strand (x-axis) was calculated. Pearson correlations between the difference in hotspot mutation frequency and predicted G4 potential were computed separately for each strand, as well as for each hotspot motif (the analyzed strand and AID hotspot are both indicated in gray above and to the side of the plot, respectively).

We next sought to determine if G4s might play a role in facilitating mutations induced by AID. Initially, we approached this by considering possible increased mutability at AID hotspots on the same strand that G4s are predicted to form. Starting from a database of mutated non-productive IGHV sequences from memory B cells we used in our previous study (30), for each available allele we calculated the mutation frequencies for each of the four distinct AID WRC hotspot motifs (AAC, AGC, TAC, TGC), separately for both strands, and then calculated the mean mutation frequency for each motif. Furthermore, we computed the difference between the mutation frequencies on either strand (top-bottom) and compared this value to the predictions made by *G4detector*. For AAC hotspots, the correlations were not significant for either strand, but were so for the other hotspots on both strands (**Figure 4**). For top strand hotspots, there were significant positive correlations between the difference in mutation frequencies and G4 potential for AGC, TAC, and TGC hotspots (**Figure 4**, bottom row). Thus, increased G4 formation potential also increases the mutation frequency of the top strand relative to the bottom strand. Similarly, the bottom strand displayed significant negative correlations for the same three hotspots, again suggesting a strand bias that correlates with G4 potential on the same strand. This result suggests that G4s may attract AID to mutate hotspots on the same strand. If we consider the top and bottom strand mutation frequencies separately (**Supplementary Table 2**), this explanation is apparent for some hotspots, for example, AGC and TGC which have significant positive correlations between top strand G4 potential and mutations on the top strand, while showing a weak correlation between top strand G4 potential and bottom strand mutations. However, for some other hotspots, for example, TAC, again considering top strand G4 potential, there appears to be a significant negative correlation with mutations on the opposite strand, for which there is no obvious mechanistic explanation.

### IGHV Alleles Display Robust G4 Potentials

**Figure 5:**
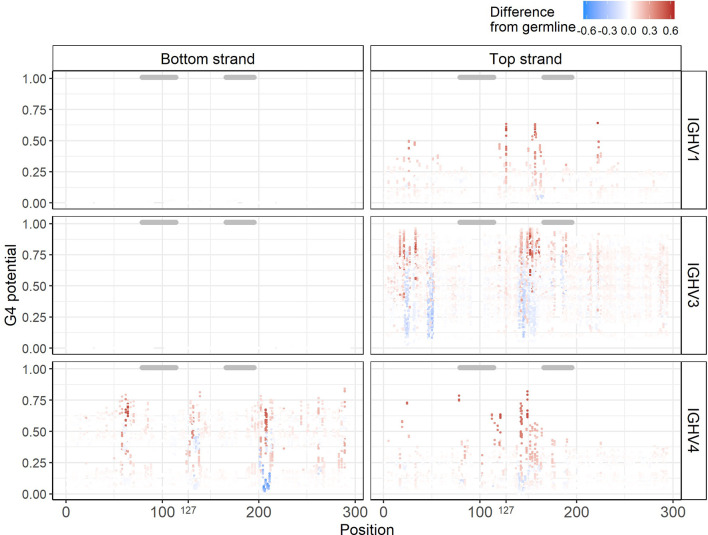
*In silico* experiments of IGHV alleles. Corresponding G4 potentials from sequences belonging to the IGHV1, IGHV3, and IGHV4 families. Each point represents one sequence containing a single mutation from its germline context at the gapped IMGT position indicated on the x-axis. The y-axis represents the G4 potential of the mutated sequence. Points are colored according to the observable difference in G4 potential in the mutated sequence from its germline. Gray bars above each plot indicate CDRs.

Having a reliable method (*G4detector*) for predicting G4s computationally enables us to perform *in silico* “experiments’’ on large numbers of hypothetically mutated sequences that might be difficult to assess experimentally using an assay such as G4-Seq. We hypothesized that mutations introducing a new G into an IGHV sequence might increase its underlying G4 potential, particularly if the mutation extends a run of G’s, for example. Conversely, mutating away a G might create a discontinuity in a stretch of G’s and result in a decrease in G4 potential. To test this hypothesis, for all 232 germline IGHV sequences, we created a new set of mutated sequences, with each newly generated sequence containing a single mutation from its germline context, and then evaluated each mutated sequence using *G4detector* (see *Methods*). We found that most mutations did not cause G4 potentials to change, as either inserting or replacing a single G base into a sequence generally did not affect much its underlying ability to form a G4, i.e. this remained within +/- 0.1 of the original value (**Figure 5**, **Supplementary Figure 4A** for IGHV1, 3, and 4 families; **Supplementary Figure 4B** for remaining IGHV families). This was particularly true of the bottom strand for the IGHV1 and IGHV3 families. In the remaining cases, however, we did observe substantial positive and negative deviations, some of which occurred in AID hotspots and coldspots (see below). In particular, the sites most susceptible to such deviations were located in G-repeat regions, as we expected. These susceptible sites also tended to be in FW regions, which should mutate less than the CDRs (indicated by gray bars above each plot) as they contribute to structural stability of the antibody (**Figure 5**). Given that single base substitutions in the IGHV alleles generally did not affect their baseline G4 potentials, this suggests that V regions are largely robust to changes in G4 structure.

As an alternative way to assess the effects of SHM on G4 potential, we again used the database of mutated IGHV sequences described above (30) to determine if SHM alters G4 potential when compared to germline (**Supplementary Figure 5**). For each IGHV gene, we computed the average G4 potential of the sequences. Again, in comparison to the germline potentials, the changes in G4 potential were mostly small, especially for the bottom strand. However, a few larger increases and decreases did occur among the mutated sequences, particularly within the IGHV4 family and to an extent within the IGHV3 family as well.

Additionally, we assessed the effect of VDJ rearrangements on G4 potential using the subset of non-productive sequences of naïve B cells in the same dataset, which should contain minimal SHM, allowing us to evaluate the rearrangement effect alone. Using *G4detector* we analyzed only the furthest 3’ section of the sequence (of length 297nt, the width of the *G4detector* input). This section includes the entire CDR3 and J gene segments, but omits most of CDR1, so is not strictly comparable to the corresponding germline values, which include all of CDR1. Considering the top strand first, for alleles of the IGHV3 family – because G4s primarily form at the 5’ end (**Figure 2A**) – we found that the CDR3 plus J gene sequence had a lower G4 potential compared to germline (**Supplementary Figure 6**). On the other hand, the mean G4 potential for the IGHV1, IGHV4, and IGHV7 families increased slightly, suggesting that the 3’ end of the rearranged V region can enhance G4 formation in these IGHV families. For the bottom strand, the effects were generally minimal. In conclusion, the J gene and CDR3, may be involved in G4 formation, but the overall effect is likely to be small.

### Mutations in AID Coldspots Lead to Decreases in G4 Potential on the Opposite Strand and Display Higher SHM When Opposite a G4

**Figure 6:**
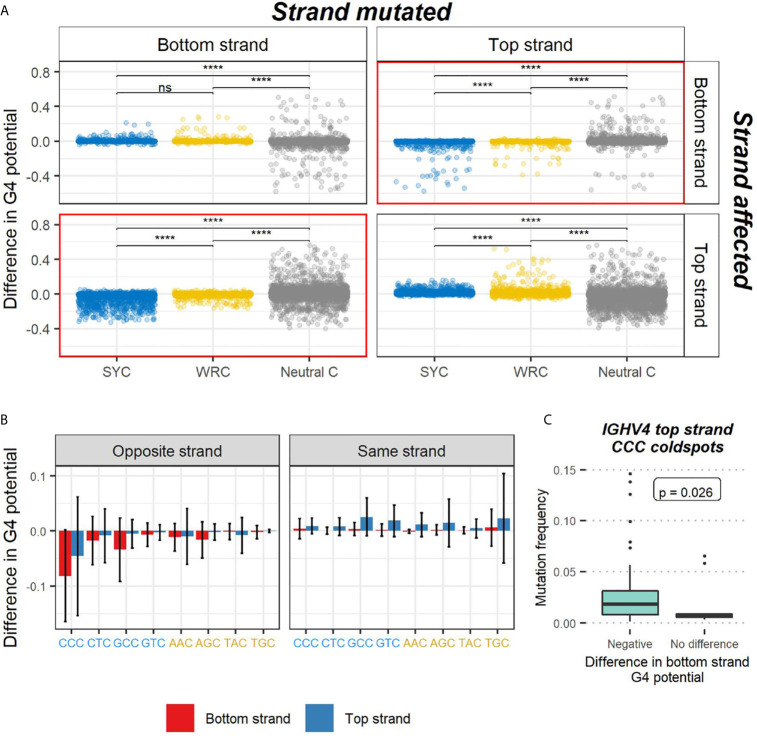
AID targeting effects on G4 potential. **(A)** Differences in G4 potential of the sequences used in the *in silico* experiments were analyzed. The plots contain sequences where the mutation occurred in C nucleotides only. Each sequence is categorized as containing a mutation in one of three AID-associated contexts: SYC coldspot, WRC hotspot, or neutral C base. Strand-specific effects on G4 potential are distinguished by having mutated either the bottom or top strand (strand mutated), and the resulting outcome taking place on either the same strand or opposite strand (strand affected). Opposite strand plots have a red border. P-values from two-sided Mann-Whitney U tests are indicated as asterisks (ns, not significant; ****p ≤ 0.0001). **(B)** Mean difference in G4 potential caused by mutations at specific SYC and WRC trinucleotide motifs. The strand subjected to mutation is indicated by the bar color (red, bottom strand; blue, top strand). The G4 potential of the strand being affected is labeled at the top of the plot in gray. The different SYC and WRC trinucleotide motifs are labeled at the bottom in blue and yellow, respectively. Error bars drawn at each bar represent -/+ 1 standard deviation. **(C)** Comparison of mutation frequencies of top strand CCC coldspots in 17 alleles from the IGHV4 family. Individual CCC motifs are separated based on the resulting outcome of the bottom strand G4 potential when mutated. The p-value of a two-sided Mann-Whitney U test comparing coldspots that negatively impacted G4 potential against those that led to no difference is reported.

We further looked to see if the findings from our *in silico* experiments were due to various AID targeting effects. AID has a preference for mutating at WRC hotspots (W=A/T, R=A/G), whereas it tends to avoid mutating at SYC coldspot motifs (S=C/G, Y=C/T). We speculated that a C>G mutation, for example, in an AID hotspot such as AGC might create tandem G’s that lead to an increased G4 potential, especially if additional G bases are located nearby. Alternatively, a mutation occurring in an AID coldspot (e.g. CCC) might, in some cases, disrupt a run of G nucleotides on the opposite strand, thus reducing that strand’s G4-forming ability. To address the consequences AID deamination might have on G4 potential, we examined only C nucleotides in our data and categorized them into three groups: SYC coldspots, WRC hotspots, and neutral C sites. In addition, we also considered strand-specific effects on G4 potentials due to a mutation taking place either in the top or bottom strand. As noted above, most mutations at C sites in general do not affect G4 potential (**Figure 5**). However, when separated by site type, the most substantial increases in G4 potential occurred in neutral C’s and WRC hotspots (**Figure 6A**). Of note, many of the prominent increases in G4 potential occurring in the top strand were driven by a single conserved top strand TGC hotspot at IMGT position 127 within the IGHV1 alleles (**Figure 5**). On the other hand, sizeable decreases in G4 potential were observed in neutral sites and SYC coldspots, and to a lesser extent, in WRC hotspots. In the event where a mutation in an SYC coldspot resulted in a drop in G4 potential in one strand, the mutation generally occurred in the opposite strand (red bordered plots in **Figure 6A**). Overall, our results display a strand-dependent disparity in G4 outcomes in relation to both AID hotspot and coldspot targeting.

We next sought to investigate the specific WRC and SYC trinucleotide motifs leading to larger differences in G4 potential. As we expected (due to complementarity with GGG), CCC coldspot mutations contributed to the largest decreases in G4 potential on the opposite strand (**Figure 6B**). We also found that GCC and GTC coldspots tended to show increases in G4 potential on the same strand (P < 10^-10^ for both GCC and GTC, one-sample Wilcoxon test). As for the shifts in G4 potential due to mutations taking place in WRC hotspots, we found top strand increases to be caused by mutations in AGC and TGC motifs on the same strand, coinciding with our expectation, given these mutations create a new GG pair. Unexpectedly we also found the same effects taking place in AAC hotspots. On the other hand, most decreases in G4 potential on one strand were due to mutations emerging from AAC and AGC hotspots on the opposite strand.

Given our general observation that mutations in top strand CCC coldspots were associated with drops in G4 potential on the bottom strand, we then assessed top strand CCC motifs in alleles belonging to the IGHV4 family, which predominantly showed high G4 potentials on the bottom strand (**Figure 1A**). Again using our previously published dataset (30), we calculated the mutation frequencies of all top strand CCC coldspots across the 17 unique IGHV4 alleles. Next, we separated those CCC motifs that when mutated, according to the *in silico* findings, either displayed: 1) no difference in bottom strand G4 potential; or 2) a negative change in bottom strand G4 potential. We found that CCC coldspots that decreased G4 potentials were targeted significantly more than those that had no impact (**Figure 6C**; Mann-Whitney U test: p-value=0.026). As for the alleles in the IGHV3 family, which displayed high top strand G4 potentials, all corresponding CCC coldspots led to decreases in G4 potential, perhaps indicating that these alleles have evolved to have their AID coldspots placed in or around G-rich areas. Overall, our results suggest that IGHV alleles that create G4s in one strand may expose coldspot motifs in the opposing strand to AID deamination, thus resulting in a relatively high mutation frequency for these particular coldspot motifs.

## Discussion

In this study, we adopted a computational approach to investigate the presence of G4s in human Ig V regions as well as the possible role these structures may have in SHM. Using *G4detector*, a pre-trained deep learning model, we assessed the G4 potential of 232 functional IGHV germline alleles. We found plausible evidence of G4s to be forming in many IGHV alleles. In particular, the IGHV3 and IGHV4 family alleles were more likely to create G4s in the top and bottom strand, respectively (**Figure 1A**). We further noted a disparity in G4 potentials across alleles belonging to the same gene (**Figure 1B**), suggesting the possibility that creating or disrupting G4-promoting regions may be subject to evolutionary pressures driving sequence variation between the IGHV alleles.

We next turned to experimental high-throughput G4 data (G4-seq) to verify the predictions made by *G4detector*. Similar to our predictions, we found G4s to be more present in the top strand than in the bottom strand (**Figure 2B**). We also found there to be a fairly strong correlation between the G4s identified by G4-seq and the predictions made by *G4detector *(**Figure 2C**), validating the accuracy of the computational predictions. In addition, we used the G4-seq data to analyze G4 activity in the switch (S) region, where G4s likely form, based on *in vitro* findings (13). In our analysis, we also found evidence of G4s assembling in the top strand of the S region (**Figure 3C**). When we qualitatively evaluated the G4 activity throughout the Ig heavy chain locus, we found the V region to be less potentially active compared to the S region (**Figure 3A** **)**. Our* *analysis reveals evidence of G4s forming in the V region, but the functional role they play in the V region most likely differs from that in the S region (17). Surprisingly, we also found a relatively high potential for G4s in the bottom strand of the constant (C) regions (**Figures 3B, C**), together with a reduced density of AGCT motifs (**Figures 3B, D**). On the other hand, the average G4 potential of the top strand of the C region was roughly equal to that of V regions on the same strand (**Supplementary Figure 3A**). We subsequently assessed the plausibility of AID being recruited directly to V region G4s. Utilizing a human IGHV repertoire dataset previously published by us (30), we analyzed the mutability across all AID WRC hotspot motifs, separately for both strands, and calculated the difference between top and bottom strand hotspot mutation frequencies. Our results revealed that there is a positive association between increased predicted G4 potential and mutational targeting of certain AID hotspots localized to the same strand (**Figure 4** and **Supplementary Table 2**). Further experimental studies need to be performed in order to validate AID-G4 binding in the Ig V region. Nevertheless, our results indicate that AID binding to G4s may occur and facilitate SHM of particular V regions, but this interaction likely occurs less frequently than in S regions. C regions mutate at very low frequency (31) and presumably do not attract AID, suggesting their G4 structures do not bind AID. The particularly low density of AGCT overlapping AID hotspots in C regions may also be a contributing factor. However, G4 structures in the C regions may serve other purposes such as transcriptional pausing (32) or control of chromatin loop extrusion (33), among many other potential mechanisms. It is unclear how AID might be excluded from these G4 structures, but since AID is clearly present at nearby loci, our results suggest that AID would need to be tightly regulated to control its movement both spatially and with strand specificity, for example, *via* tight coupling with transcriptional elongation (34).

Having a reliable tool to computationally assess G4 potential in the IGHV alleles enabled us to perform *in silico* experiments. We began by generating single base substitutions in the IGHV sequences, and then running the mutated sequences through *G4detector* to find how either inserting or replacing a G nucleotide changed the G4 potential of the germline context. Inserting a mutation generally had no impact on the G4 potential of a sequence (**Figure 5**), suggesting that the underlying G4 potential of the IGHV alleles are largely robust to mutation. We did, however, observe some substantial deviations, both positive and negative, which tended to occur in FW regions. Given that FW regions are typically mutated less, compared to CDRs, and that mutations in FW regions are predicted to mostly reduce the probability of G4 formation, we speculate that the ability to maintain G4s in IGHV alleles may have co-evolved together with SHM potential. For example, transcriptional stalling of RNA Pol II might occur if G4s are present on the template strand (35, 36). As a result, most IGHV alleles (with the exception of IGHV4) may have evolved to reduce G4s on the template strand, by avoiding mutations in areas that might raise the G4 potential, as a way of ensuring transcription would proceed unhindered. In the case of the IGHV4 family, pausing in FW regions adjacent to a CDR may lead to more CDR mutations as a consequence of, for example, negative supercoiling in the wake of the transcription bubble (37). Antisense transcription (38, 39) may similarly interact with top strand G4s. Note that in our experimental *in silico* approach, we only considered single base substitutions. However, in future work one might examine combinations of two or more mutations to understand possible mutational interactions with respect to G4s, and the possible co-evolution between SHM and G4 structures. Due to the large number of combinatorial possibilities, if we were to consider realistic levels of SHM (>10 mutations), such an analysis is currently computationally prohibitive, so we leave it for possible future work.

We have evaluated the relationship between G4s and SHM by analyzing the mutations in our simulations and mapping them to AID-associated motifs. We hypothesized that mutations in canonical AID hotspots, defined by WRC motifs, might increase G4 stability by directly creating tandem G nucleotides (e.g. AGC > AGG); alternatively, a mutation at an AID coldspot such as CCC might compromise G4 potential since this could create a discontinuity in a G-repeat located in the opposite strand (e.g. CCC/GGG > CCD/HGG; where D=A/G/T and H=A/C/T). Our analysis showed that mutations in AID hotspots often led to modest increases in the G4 potential of the same strand where the mutation occurred (**Figure 6A**). On the other hand, mutations in AID coldspots taking place in one strand generally decreased G4 potential of the opposite strand (**Figure 6A**), again supporting our hypothesis. We then analyzed individual AID hotspot and coldspot motifs to see which particular motifs were responsible for driving these changes. We found that mutations in CCC coldspots accounted for the largest decreases in G4 potential of the opposing strand (**Figure 6B**). Also, increases in G4 potential were generated by mutations in both AID hotspots as well as coldspots. Since we found that mutations in CCC coldspots were predicted to reduce G4 potential, we utilized the same high-quality repertoire dataset to analyze SHM in these motifs. Specifically, we considered alleles from the IGHV4 family and evaluated whether mutations in top strand CCC coldspots leading to decreased G4 potential in the bottom strand were targeted differently than those that did not alter the germline potential when mutated. We found that CCC coldspots predicted to reduce the G4 potential (thus likely opposite a GGG that is part of a G4) were, in fact, targeted significantly more (**Figure 6C**), further suggesting that a G4 formed in the bottom strand may increase exposure of the corresponding subregion on the top strand to AID. Future studies using more specific assays, for example, involving V genes where the G4 potential has been mutated away, may be able to establish more precisely if and when AID binds to G4 structures in V regions, and how frequently G4s form in the V region and their potential effect on SHM. It is also unknown, yet feasible, that other secondary structures, such as cruciform DNA (40) and intercalated-motifs (41), are also involved and contribute to SHM.

## Data Availability Statement

The original contributions presented in the study are included in the article/**Supplementary Material**. Further inquiries can be directed to the corresponding author.

## Author Contributions

CT and TM designed the research, performed the data analysis and prepared the manuscript. All authors contributed to the article and approved the submitted version.

## Funding

This study is supported by the multi-PI NIH grant 1R01AI132507-01A1.

## Conflict of Interest

The authors declare that the research was conducted in the absence of any commercial or financial relationships that could be construed as a potential conflict of interest.
