# Supplementary material for: Characterization of DNA G-Quadruplex Structures in Human Immunoglobulin Heavy Variable (IGHV) Genes
Source: Front Immunol. 2021 May 10;12:671944. doi: 10.3389/fimmu.2021.671944 (PMC8141862; doi:10.3389/fimmu.2021.671944)
Supplement: Supplementary file 1 [file Presentation_1.pptx]

## Slide 1
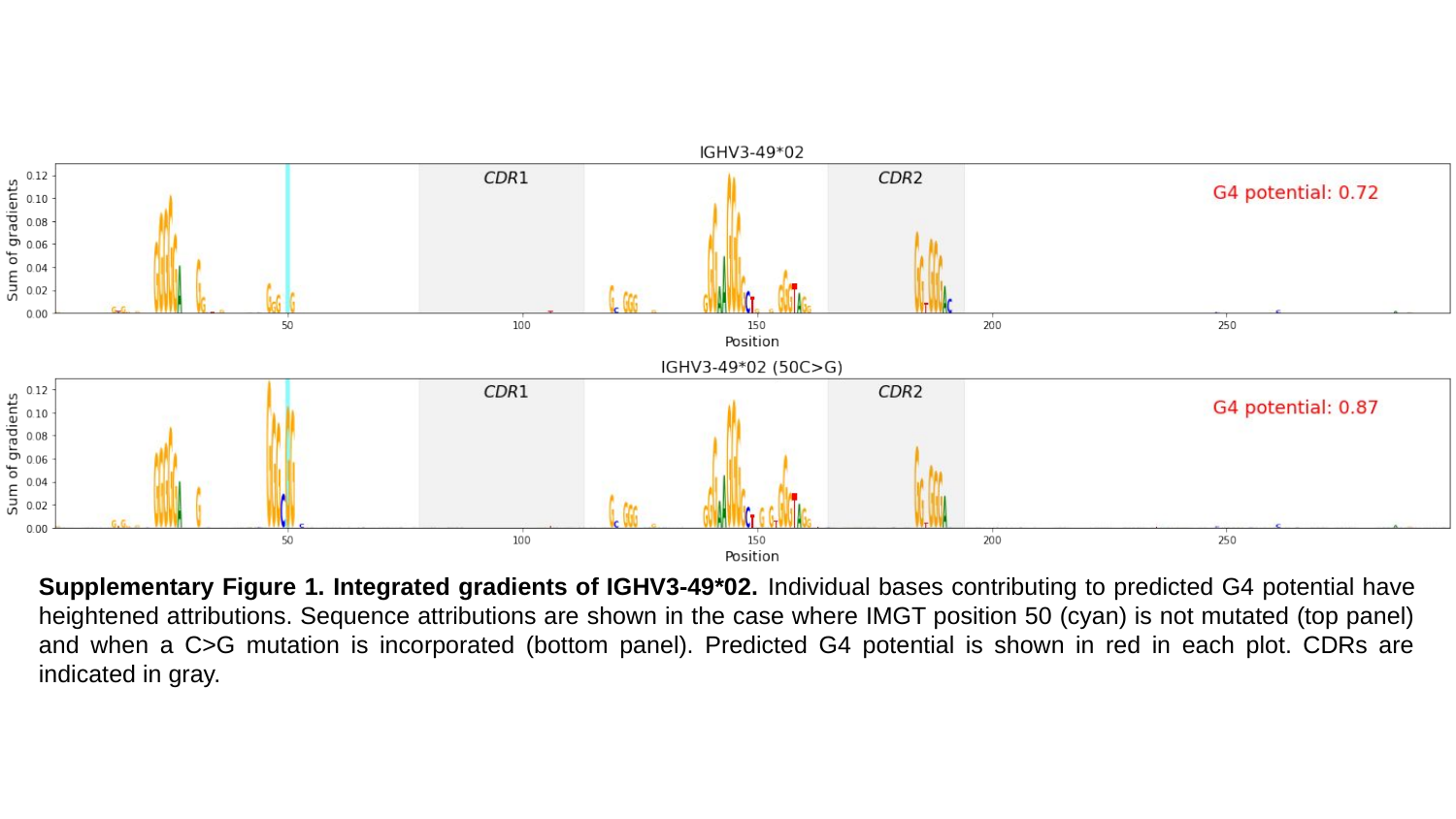

Supplementary Figure 1. Integrated gradients of IGHV3-49*02. Individual bases contributing to predicted G4 potential have heightened attributions. Sequence attributions are shown in the case where IMGT position 50 (cyan) is not mutated (top panel) and when a C>G mutation is incorporated (bottom panel). Predicted G4 potential is shown in red in each plot. CDRs are indicated in gray.

## Slide 2
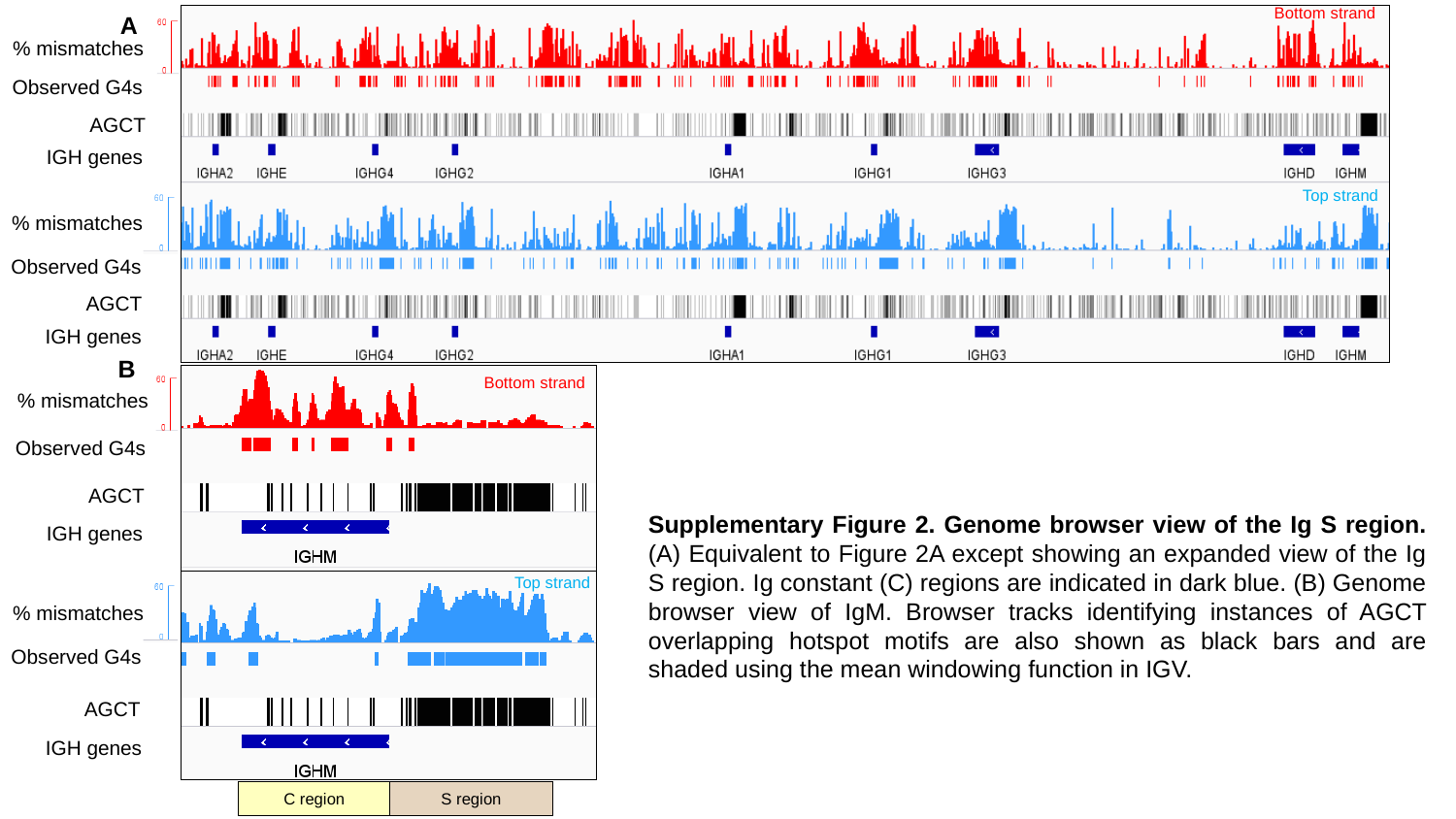

Bottom strand
A
% mismatches
Observed G4s
AGCT
IGH genes
Top strand
% mismatches
Observed G4s
AGCT
IGH genes
B
Bottom strand
% mismatches
Observed G4s
AGCT
Top strand
% mismatches
Observed G4s
AGCT
C region
S region
Supplementary Figure 2. Genome browser view of the Ig S region. (A) Equivalent to Figure 2A except showing an expanded view of the Ig S region. Ig constant (C) regions are indicated in dark blue. (B) Genome browser view of IgM. Browser tracks identifying instances of AGCT overlapping hotspot motifs are also shown as black bars and are shaded using the mean windowing function in IGV.
IGH genes
IGH genes

## Slide 3
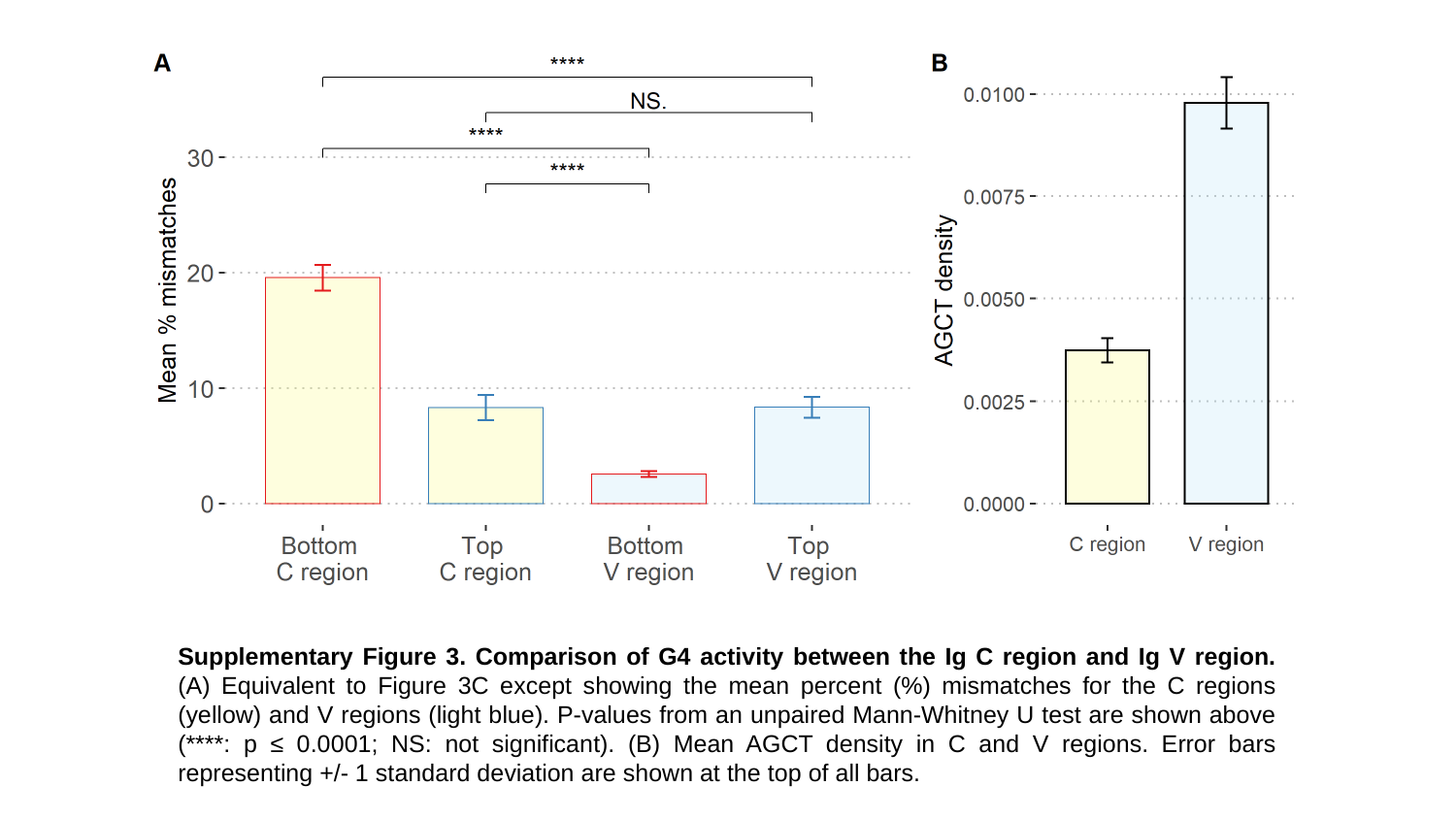

Supplementary Figure 3. Comparison of G4 activity between the Ig C region and Ig V region. (A) Equivalent to Figure 3C except showing the mean percent (%) mismatches for the C regions (yellow) and V regions (light blue). P-values from an unpaired Mann-Whitney U test are shown above (****: p ≤ 0.0001; NS: not significant). (B) Mean AGCT density in C and V regions. Error bars representing +/- 1 standard deviation are shown at the top of all bars.

## Slide 4
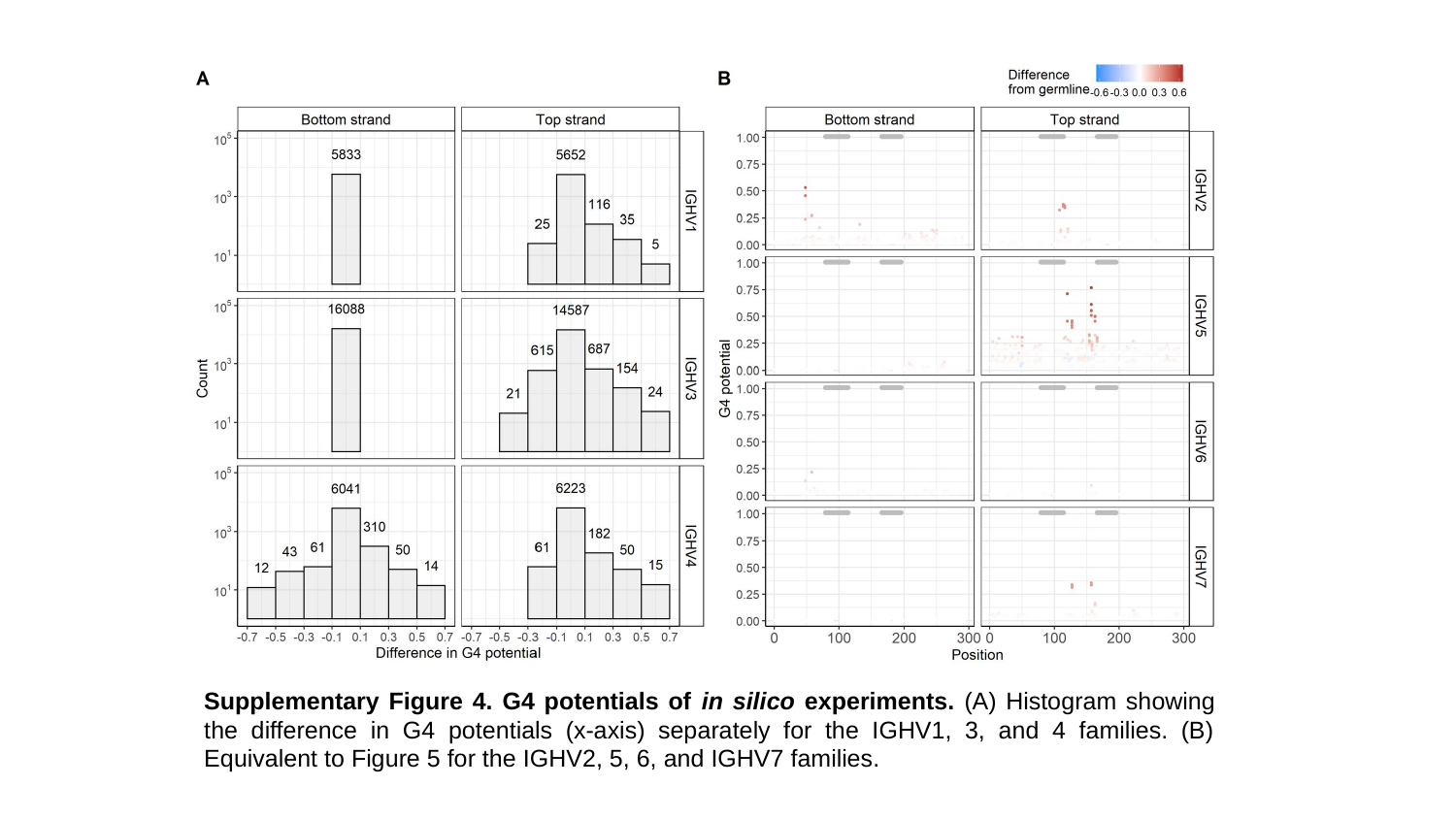

Supplementary Figure 4. G4 potentials of in silico experiments. (A) Histogram showing the difference in G4 potentials (x-axis) separately for the IGHV1, 3, and 4 families. (B) Equivalent to Figure 5 for the IGHV2, 5, 6, and IGHV7 families.

## Slide 5
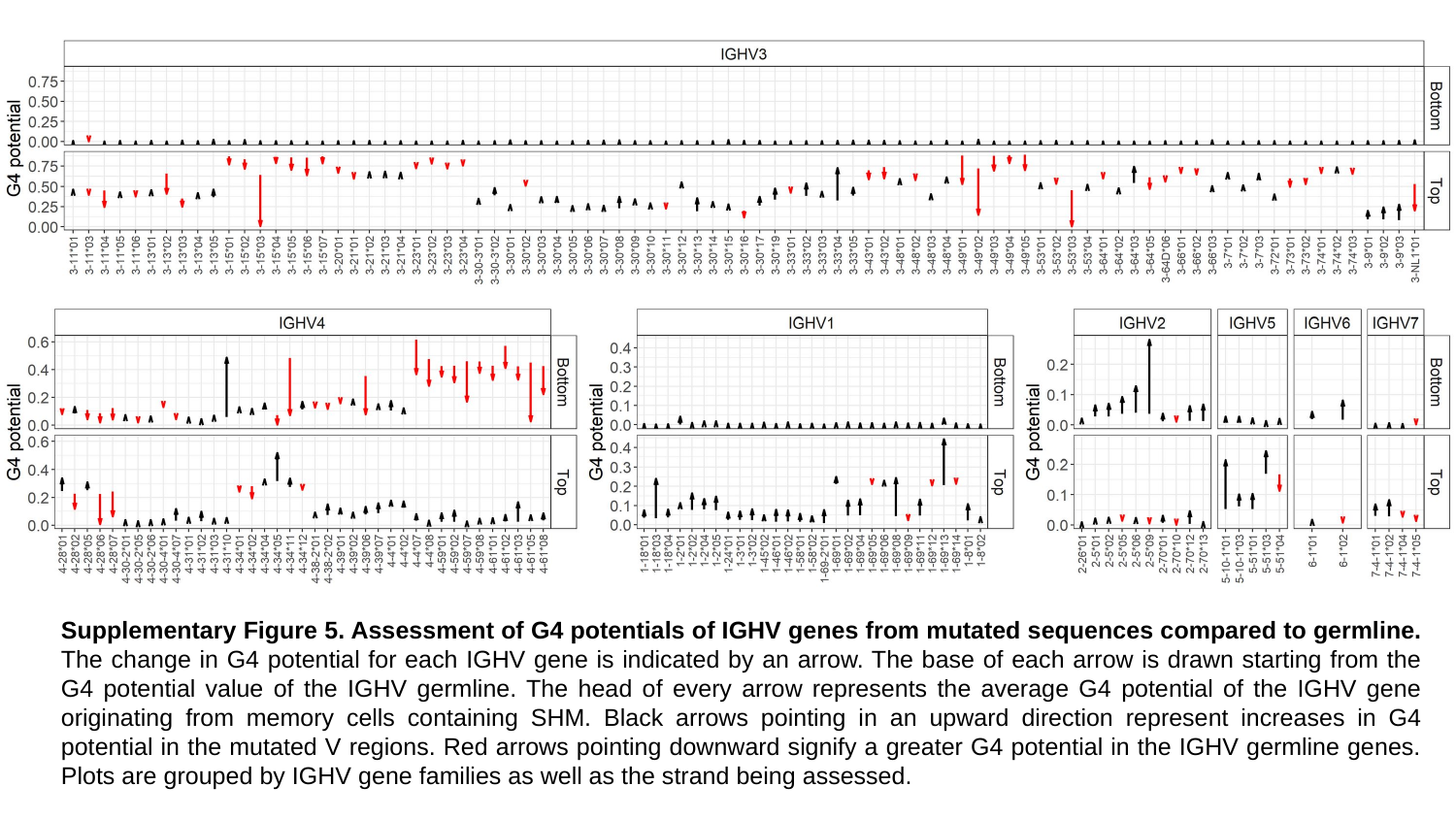

Supplementary Figure 5. Assessment of G4 potentials of IGHV genes from mutated sequences compared to germline. The change in G4 potential for each IGHV gene is indicated by an arrow. The base of each arrow is drawn starting from the G4 potential value of the IGHV germline. The head of every arrow represents the average G4 potential of the IGHV gene originating from memory cells containing SHM. Black arrows pointing in an upward direction represent increases in G4 potential in the mutated V regions. Red arrows pointing downward signify a greater G4 potential in the IGHV germline genes. Plots are grouped by IGHV gene families as well as the strand being assessed.

## Slide 6
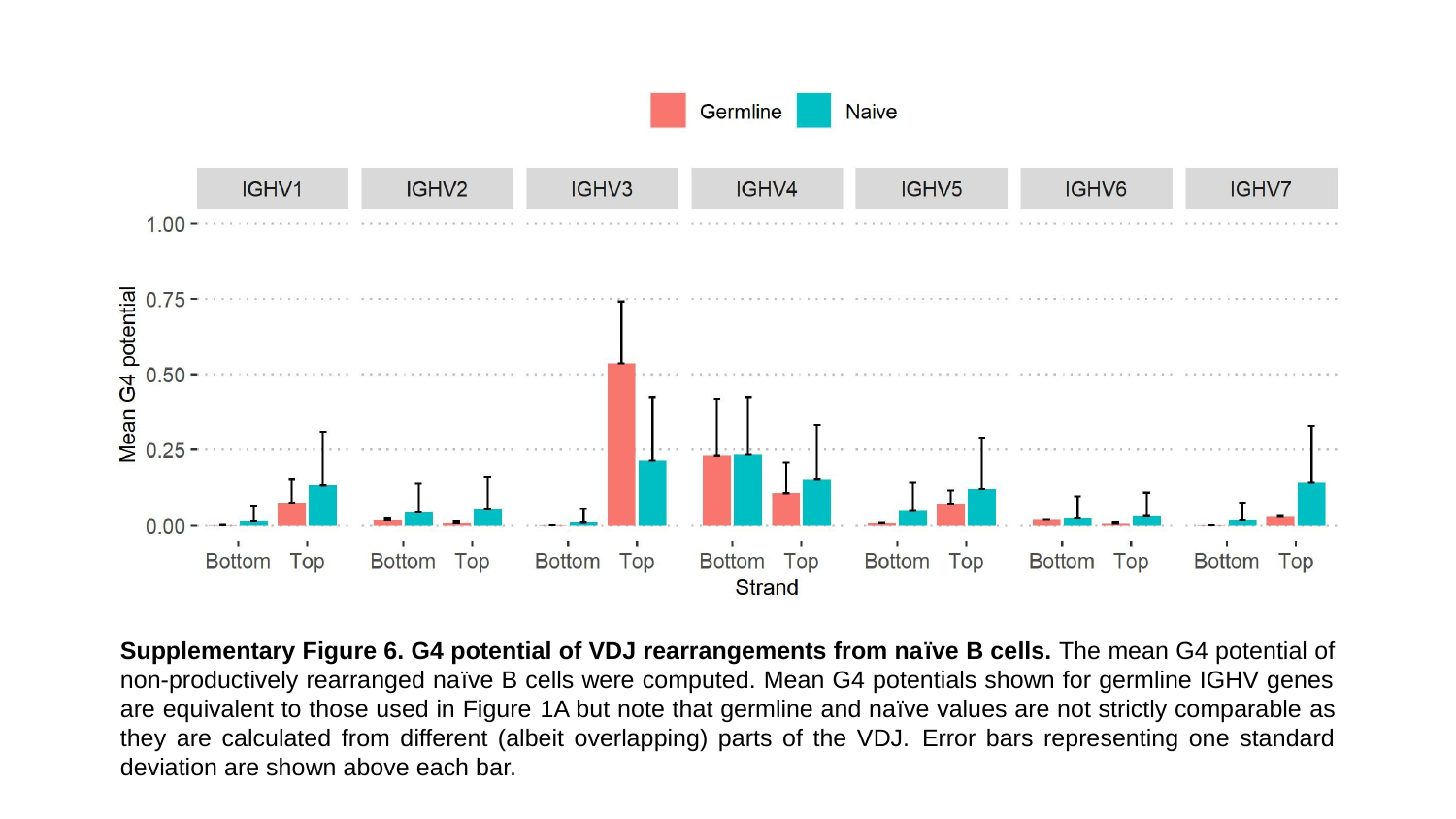

Supplementary Figure 6. G4 potential of VDJ rearrangements from naïve B cells. The mean G4 potential of non-productively rearranged naïve B cells were computed. Mean G4 potentials shown for germline IGHV genes are equivalent to those used in Figure 1A but note that germline and naïve values are not strictly comparable as they are calculated from different (albeit overlapping) parts of the VDJ. Error bars representing one standard deviation are shown above each bar.
